# Monitoring Therapeutic Treatments against *Burkholderia* Infections Using Imaging Techniques

**DOI:** 10.3390/pathogens2020383

**Published:** 2013-05-23

**Authors:** Tiffany M. Mott, R. Katie Johnston, Sudhamathi Vijayakumar, D. Mark Estes, Massoud Motamedi, Elena Sbrana, Janice J. Endsley, Alfredo G. Torres

**Affiliations:** 1 Department of Microbiology and Immunology, University of Texas Medical Branch, Galveston, TX 77555, USA; E-Mails: tmmott@utmb.edu (T.M.M.); rkjohnst@utmb.edu (R.K.J.); suvijaya@utmb.edu (S.V.); elsbrana@utmb.edu (E.S.); jjendsle@utmb.edu (J.J.E.); 2 Department of Infectious Diseases, College of Veterinary Medicine, University of Georgia, Athens, GA 30602, USA; E-Mail: dmestes@uga.edu; 3 Center for Biomedical Engineering, University of Texas Medical Branch, Galveston, TX 77555, USA; E-Mail: mmotamed@utmb.edu; 4 Department of Pathology, University of Texas Medical Branch, Galveston, TX 77555, USA

**Keywords:** *Burkholderia mallei*, CpG, immunomodulation, *in vivo* imaging, infrared fluorescent imaging

## Abstract

*Burkholderia mallei*, the etiologic agent of glanders, are Category B select agents with biothreat potential, and yet effective therapeutic treatments are lacking. In this study, we showed that CpG administration increased survival, demonstrating protection in the murine glanders model. Bacterial recovery from infected lungs, liver and spleen was significantly reduced in CpG-treated animals as compared with non-treated mice. Reciprocally, lungs of CpG-treated infected animals were infiltrated with higher levels of neutrophils and inflammatory monocytes, as compared to control animals. Employing the *B. mallei* bioluminescent strain CSM001 and the Neutrophil-Specific Fluorescent Imaging Agent, bacterial dissemination and neutrophil trafficking were monitored in real-time using multimodal *in vivo* whole body imaging techniques. CpG-treatment increased recruitment of neutrophils to the lungs and reduced bioluminescent bacteria, correlating with decreased bacterial burden and increased protection against acute murine glanders. Our results indicate that protection of CpG-treated animals was associated with recruitment of neutrophils prior to infection and demonstrated, for the first time, simultaneous real time *in vivo* imaging of neutrophils and bacteria. This study provides experimental evidence supporting the importance of incorporating optimized *in vivo* imaging methods to monitor disease progression and to evaluate the efficacy of therapeutic treatment during bacterial infections.

## 1. Introduction

*Burkholderia mallei* are Gram-negative, non-motile, facultative intracellular pathogens that cause a disease known as glanders. Primarily associated with solipeds (horses, mules, *etc*.), glanders-causing bacteria can be transmitted via highly infectious respiratory or cutaneous secretions to humans, occasionally felidae, and other susceptible species [1,2,3,4,5,6]. Despite reduced disease incidence through national control programs and veterinary intervention, glanders remains endemic in various areas of the Middle East, Asia, Africa and South America [1,4,5,6,7]. In humans, depending on strain virulence, route of infection and host susceptibility, a presumed low infectious dose causes an array of symptoms ranging from asymptomatic acquisition to life- threatening pneumonia and bacteremia [8,9,10,11,12]. As high mortality rates can be reached in untreated infections and no successful therapy has been developed, *B. mallei* remains as a top candidate for bioterrorist use and thus has been classified as a category B biothreat agent [13]. Despite the importance of this pathogen, very limited studies have been performed to understand the bacterial pathogenic mechanisms associated with disease. Further, another limiting factor is that there is no reliable vaccine against glanders.

In developing vaccines, researchers have looked to the employment of adjuvants to generate and/or improve protective immunity. There has been significant interest in using cytosine-phosphate-guanine (CpG)-containing oligodeoxynucleotides (ODNs) as an adjuvant for preventative therapeutic measures [14,15,16,17,18,19,20,21,22,23,24,25,26]. CpG provides protection from a variety of pathogens, such as *Klebsiella pneumoniae, Yersinia pestis, Listeria monocytogenes, Burkholderia pseudomallei*, respiratory syncytial virus and human immunodeficiency virus, by enhancing non-specific immunity commonly associated with a strong polarized Th1-cell response, including increased production of IFN-γ and Th1-associated antibody isotypes [20,21,22,23,24,25,26]. Pre- and post-vaccination with CpG has been shown to protect and in some cases may contribute to the cessation of disease transmission [20]. Mimicking bacterial DNA, CpG ODNs activate toll-like receptor (TLR) 9 signaling and thus, function as potent stimulators of innate immunity [27,28]. CpG ODNs are categorized in A-, B- and C-classes, based on varying properties of length, sequence, backbone and formation of secondary or tertiary structures. Although all act as TLR-9 agonists, the three classes have been described as eliciting different innate immune responses [28]. The class-A is characterized by strong natural killer (NK) cell and precursor dendritic cell (pDC) activation, high levels of IFN-α production, and limited B cell activation, whereas the class-B is categorized by strong B cell activation, moderate NK and pDC activation with moderate IL-12 and limited IFN-α production [29,30,31,32,33,34]. The class-C has intermediate properties of both A- and B-class and thus is categorized by a strong B cell, antigen presenting cells (APC) and NK cell activation, induction of pDC IFN-α production and preferential development and differentiation of T helper 1 (Th1) cells [27,33,35,36]. 

The activation of innate immunity by the administration of CpG has been shown to provide protection against an array of intracellular pathogens [20,21,22,23,24,25,26]. Against low aerosol challenge of *B. mallei*, BALB/c mice pre-treated with CpG had lower levels of bacterial burden in the lungs and increased survival compared to controls [17]. The protection provided by CpG pre-treatment was associated with enhanced levels of IL-12 and IFN-γ, IFN-γ-inducible protein 10 and IL-6 [17]. Similar results were seen in an acute fatal sepsis model of *B. pseudomallei* infection in BALB/c mice where CpG pre-treatment conferred more than 90% protection which was attributed to elevated levels of IL-12 and IFN-γ [18]. In an attempt to define immune correlates of protection provided by CpG pre-treatment, recent studies have shown that protection against an acute respiratory model of *B. pseudomallei* infection in BALB/c mice is linked to elevated levels of IL-12 and recruitment of inflammatory monocytes and neutrophils into the lungs prior to infection [15]. Because *B. mallei* is considered a dwarfed clone of *B. pseudomallei*, data from experiments ascertaining the protective efficacy of CpG treatment in an acute model of glanders should reflect the infectious trends that of those seen in melioidosis [37].

As this information is lacking, in the present study, the protective potential of CpG pre-treatment was evaluated in an acute respiratory BALB/c mouse model of *B. mallei* infection. In addition to assessing class-C CpG ODN protective capabilities and the role inflammatory cell recruitment is playing during murine glanders, our study has focused on the implementation of *in vivo* imaging techniques to monitor disease progression and treatment. Since our previous study showed that CpG class-C provides the greatest protection against *B. pseudomallei*, this CpG ODN was chosen for evaluation. The bioluminescent *B. mallei* reporter strain CSM001 was used to monitor real time bacterial infection combined with a new technique that employs a Neutrophil-Specific Fluorescent Imaging Agent to visualize neutrophil trafficking *in vivo*.

## 2. Results and Discussion

### 2.1. Class-C CpG ODNs Treatment Increases Percent Survival in BALB/c Mice

**Figure 1:**
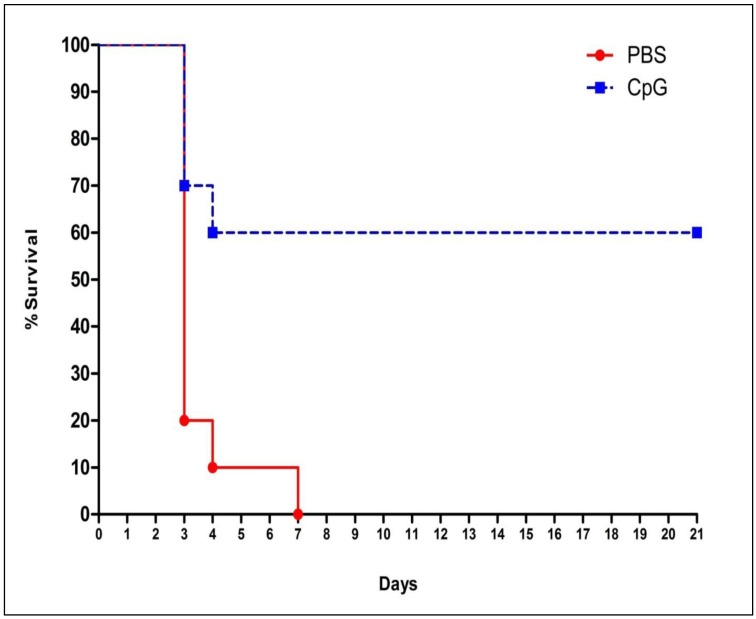
Survival of BALB/c mice immunized with Class-C CpG or PBS and challenged with *B. mallei* CSM001. Both a Log-rank test (*p =* 0.0029) and a Gehan-Breslow-Wilcoxon test (*p =* 0.0094) found both Kaplan-Meier survival curves to be statistically different.

Using the pre-established acute respiratory challenge (*i.e.*, intranasal) model of glanders [38], two groups of 10 BALB/c mice were treated intranasal (i.n.) with 20 µg of class-C CpG or PBS. Twenty four hours after treatment, BALB/c mice were infected i.n with 10^4^ cfu of *B. mallei* CSM001. This combination of class-C CpG ODN treatment and CSM001 challenge was used in all the following studies unless otherwise mentioned. As previously demonstrated with this model, the majority of deaths in the control group occurred on days 3 and 4, with all PBS-treated animals succumbing to infection on day 7 (Figure 1). 

On the other hand, treatment with class-C CpG resulted in increased protection shifting the curve from 0% to 60% survival until the study was terminated at day 21. 

### 2.2. Class-C CpG ODNs Treatment Reduces Bacterial Load in BALB/c Mice

**Figure 2:**
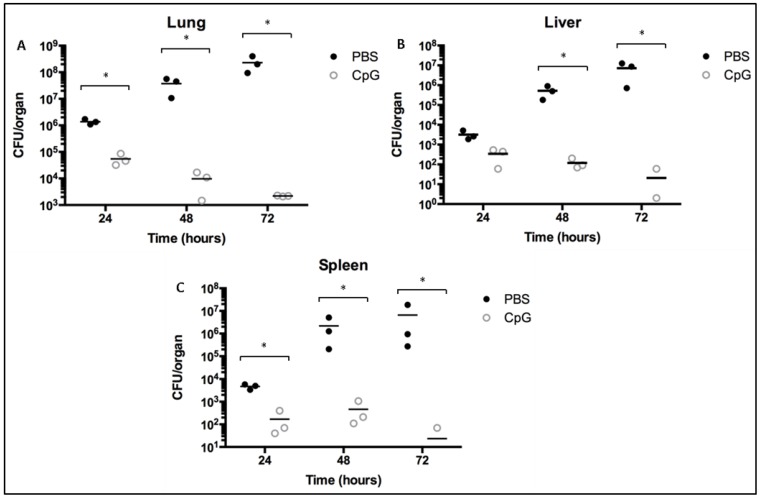
Bacterial burden of BALB/c mice immunized with Class-C CpG (open circles) or PBS (solid circles) and challenged with *B. mallei* CSM001. Bacterial burden in the lungs (**A**), liver (**B**) and spleen (**C**) was determined in three mice per group per time point. To determine statistical differences, values were log transformed and then subjected to a paired two-tailed Student t test. Statistically significant value is designated * *p <* 0.05.

To assess the effects of class-C CpG ODN treatment on CSM001’s ability to establish infection and colonize target organs, BALB/c mice were treated and infected as described in the experimental section. At 24, 48 and 72 h post-infection, three animals per group were euthanized, their lungs, livers and spleens homogenized, serial diluted in PBS and plated on LBG agar supplemented with kanamycin (Km). BALB/c mice treated with class-C CpG ODN showed significantly reduced levels of bacteria in the lung, liver and spleen at every time point, as compared to PBS-treated BALB/c mice (Figure 2).

Bacterial numbers peaked at mean values of 5.47 × 10^4^ cfu, 3.43 × 10^2^ cfu and 1.70 × 10^2^ cfu in the lung, liver and spleen, respectively; in class-C CpG ODN treated mice at 24 h and then receded to low levels in the lung and low or undetectable levels in the liver and spleen by 72 h post-infection. On the other hand, bacterial numbers in PBS-treated mice continued to increase exponentially over the sampling period peaking at 72 h with a mean value of 2.31 × 10^8^ cfu, 7.23 × 10^6^ cfu and 6.60 × 10^6^ cfu in the lung, liver and spleen, respectively.

### 2.3. Class-C CpG ODNs Treatment Leads to Higher Neutrophil-Specific Fluorescence Signal in BALB/c Mice

**Figure 3:**
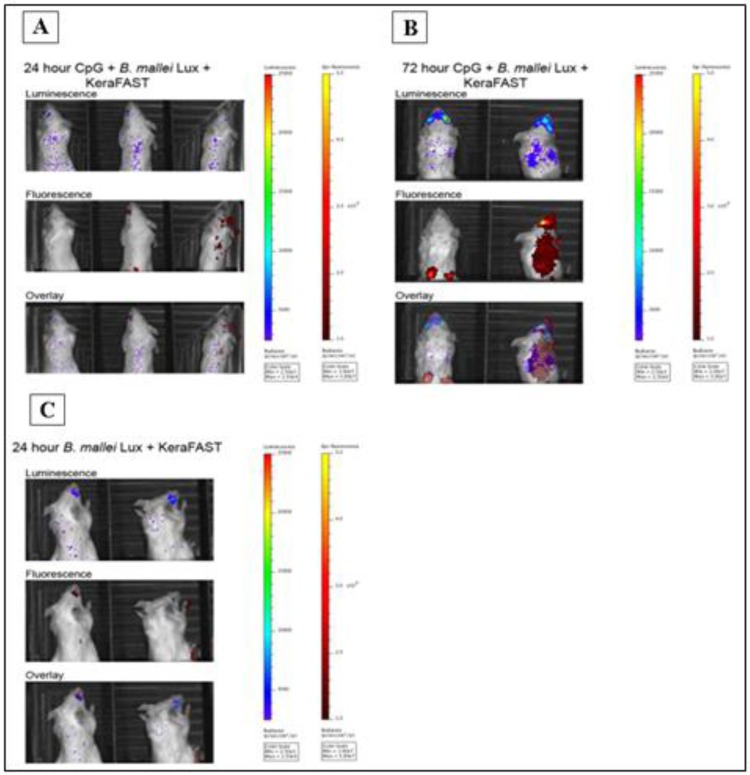
*In vivo* whole body imaging of BALB/c mice immunized with Class-C CpG or PBS and challenged with *B. mallei* CSM001. *In vivo* whole body bioluminescence and fluorescence images of Class-C CpG treated (**A**,**B**) and PBS treated (**C**) were taken at 24 h (A,C) and 72 h (B) post-infection. The intensity of emission is represented as a pseudocolor image. The luminescence and fluorescent images were then overlaid to visualize bacteria and neutrophil localization. (Overlay)

As previously shown for *B. pseudomallei*, class-C CpG ODNs treatment resulted in increased neutrophil and inflammatory monocyte trafficking to the lung, reduced bacterial burden and increased survival time compared to BALB/c mice treated with PBS [15]. Due to the close relatedness of these two pathogens, we then focused on the visual analysis of neutrophil trafficking in BALB/c mice treated with class-C CpG ODN or PBS and infected with *B. mallei* CSM001. Three hours prior to imaging, two BALB/c mice from each group were administered the Neutrophil-Specific, Fluorescent Imaging Agent via tail vein injection. At 24, 48 and 72 h, BALB/c mice were anesthetized and monitored for bioluminescent bacteria and fluorescent neutrophil-specific signal, using an *in vivo* imaging system (IVIS Spectrum) to collect and quantifying the photons emitted by neutrophils and CSM001 within the animals (Figure 3). 

Nothing significant was visualized at 24 h (Figure 3A,C) or 48 h (data not shown); however, at 72 h, *B. mallei* CSM001 organisms are seen in the head region, lungs, livers and spleens of CpG treated mice. When looking at neutrophil trafficking and recruitment to the site of infection, at the 72 h (Figure 3B) time points, neutrophils and CSM100 were imaged simultaneously, supporting the value of *in vivo* imaging technology to study progression of bacterial infection and host responses to combat and control bacterial dissemination. PBS-treated animals expired before the 72 h and thus were not imaged.

### 2.4. Class-C CpG ODNs Treatment Leads to Increase Levels of Inflammatory Monocytes during Early Infection of BALB/c Mice

**Figure 4:**
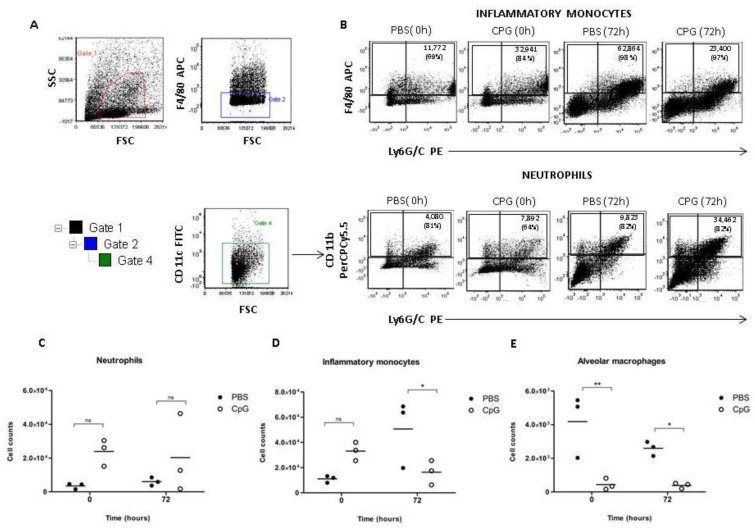
Flow cytometry analysis of inflammatory cell populations in BALB/c mice immunized with Class-C CpG or PBS and challenged with *B. mallei* CSM001. Cells from the lung were isolated at 0 h (before infection) and 72 h (after infection). Cells were labeled with Ly6G/C-PE, CD11c-FITC, CD11b-PerCPCy5.5, F4/80-APC monoclonal antibodies or corresponding isotype controls and analyzed using FCS Express v4.0 Flow Research Edition (De Nova Software). Panel A (Gate1) shows gating of leukocytes from PBS treated group by selecting cells that display the appropriate forward and side scatter characteristics. The inflammatory monocyte population (F4/80+ Ly6G/C+) as depicted in panel.4B (upper plots) were obtained from cells in gate 1. Cell populations were further separated based on lack of the F4/80 marker (gate 2) and lack of the CD11c (gate 4) in order to obtain the neutrophil population (CD11c, F4/80-, CD11b+, Ly6G/C+) as shown in panel B (lower plots). The percentages in Panel 4B represent the percentage of inflammatory monocytes from the F4/80+ population and neutrophils from the CD11b+ population, as indicated by the box in the upper quadrants of the flow plots in 4B. Results are representative of three animals per group. Shown in panels C, D and E are neutrophil, inflammatory monocytes and alveolar macrophage numbers respectively of PBS (solid circles) or Class-C CpG (open circles) pre-treated animals at 0 h and 72 h after infection. Data are shown as mean ± SEM. One-way ANOVA followed by a Dunnett’s multiple comparison tests for group comparisons (Graph Pad Software v4.0). Statistically significant value is designated * *p <* 0.05.

The influx of neutrophils, inflammatory monocytes and alveolar macrophage populations associated with class-C CPG ODNs treatment and *B. mallei* infection was assessed by flow cytometry of cells found in lung tissue. As shown in Figure 4, a hierarchical gating strategy was to quantitate numbers of specific populations within lung tissue based on surface marker expression.

Acquired samples were first selected based on side scatter and forward scatter characteristics consistent with leukocytes (Gate 1) (Figure 4A). The Gate 1 cell populations were further separated based on expression of F4/80 (Gate2, F480+ and Gate 3, F4/80–) to distinguish monocyte and macrophage populations (F4/80+) from neutrophils (F4/80–). Neutrophil numbers were further determined from the F4/80- population based on lack of CD11c expression (Gate 4) and expression of CD11b (Gate 5) and Ly6 G/C (Figure 4b). Data is shown as number of events (cells) from the same volume of total lung homogenate as described in the experimental section. The use of these markers additionally enabled assessment of alveolar macrophages which are phenotypically defined as F4/80+CD11b–CD11c+. 

Twenty four hours (Time 0 h) after pre-treatment with class-C CpG ODN, the numbers of inflammatory monocytes (F4/80+Ly6 G/C+) increased in BALB/c mice compared with those treated with PBS (Figure 4D). At 72 h, an inverse trend is seen with PBS-treated mice showing significantly higher numbers of inflammatory monocytes (Figure 4D). Although neutrophil levels were 1.7 times higher at 0 h in class-C CpG ODN treated *vs.* PBS treated BALB/c mice, this increase was not statistically significant (Figure 4C). Following challenge with *B. mallei*; however, neutrophil numbers were increased in both treatment groups by 72 h post-infection. PBS-treated mice had significantly higher numbers of alveolar macrophages numbers at 0 and 72 h compared to class-C CpG ODN treated mice (Figure 4E).

### 2.5. Class-C CpG ODNs Treatment Results in Decreased Tissue Damage in the Lungs

**Figure 5:**
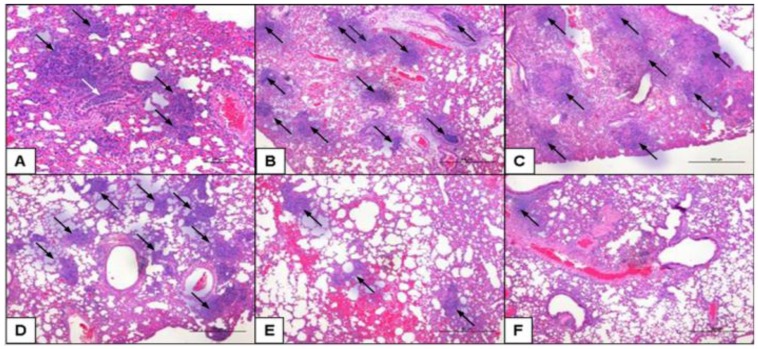
Histological analysis of lungs from BALB/c mice immunized with Class-C CpG or PBS and challenged with *B. mallei* CSM001. Representative images of Hematoxylin and Eosin stained mouse lungs sections from *B. mallei*-infected mice pre-treated with PBS (**A**–**C**) or class-C CpG ODN (**D**–**F**) at 24 (A,D), 48 (B,E) and 72 h (C,F) after challenge. Magnification 40x and scale bar = 100µM. Each panel is representative of the tissue pathology observed in three mice per treatment group.

Lungs sections from *B. mallei*-infected BALB/c mice treated with class-C CpG ODN or PBS were compared at 24, 48 and 72 h after challenge (Figure 5). 

**Supplemental Figure 1:**
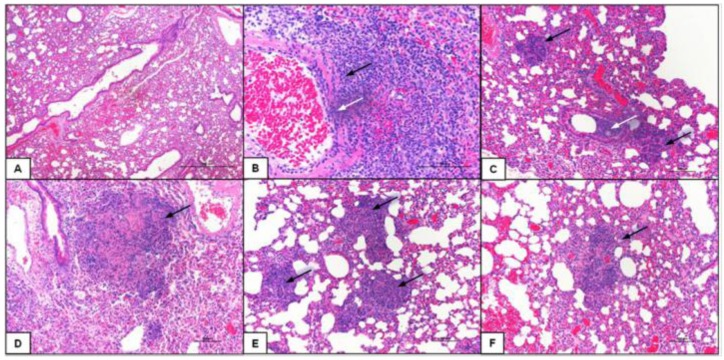
Representitve examples of lung tissue pathology seen in infected BALB/c mice. Representative images of Hematoxylin and Eosin stained mouse lungs sections that highlight the following pathology: normal lung (**A**); Perivasculitis (black arrow) and vasculitis with neutrophil component (white arrow) (**B**); scattered microabscesses (black arrows) and cell debris in bronchial lumen with neutrophil component (white arrow) (**C**); areas of necrosis (black arrows) (**D**); microabscesses (black arrows) (**E**); and small necrotic foci (black arrow) (**F**); Magnification 40× and scale bar = 100µM.

At 24 h, lungs from class-C CpG ODN- and PBS-treated BALB/c mice show mild to moderate and moderate to focally severe, respectively, perivascular and peribronchial inflammation with a considerable neutrophilic component. Interstitial inflammatory infiltrates which are mostly neutrophils are present with numerous scattered micro-abscesses (black arrows) throughout the lung parenchyma (Figure 5A,D). Collection of neutrophils and cellular debris within the bronchial lumen (white arrow) are also seen in PBS-treated BALB/c mice (Figure 5A and supplemental Figure 1B). At 48 and 72 h, areas of necrosis begin to form in microabscesses (black arrows) in both treatment; although, necrotic areas in the lungs of CpG treated BALB/c mice (Figure 5E,F and supplemental Figure 1D,E) are much smaller and localized compared to lungs of PBS treated BALB/c mice (Figure 5B,C). In addition, PBS-treated mice displayed groups of neutrophils and cellular debris within the bronchial lumen (supplemental Figure 1C) not seen in class-C CpG ODN treated mice. 

### 2.6. Discussion of Results

*B. mallei* isolates are a causative agent of glanders, a disease with high mortality rates in untreated infections [13]. Despite *B. mallei’s* classification as a biothreat agent, there is currently no licensed pre- and post-exposure vaccine that may protect against infection*.* There are very little data on the efficacy of treating human glanders with antibiotics. Currently, the treatment regimen consists of long-term administration of mixed antibiotics that, if diagnosed early and accurately, is often only partially effective [9]. In the case of *B. pseudomallei* and *B. mallei*, there has been significant interest in using the cytosine-guanine (CpG)-containing oligodeoxynucleotides (ODNs) as adjuvant for preventative therapeutic measures [14,15,16,17,18,19,31,39,40,41]. The interest resides in the fact that CpG interacting with TLR-9 receptors, primarily expressed on B cells and DCs, stimulates the secretion/production of Ig, cytokine and chemokines and biases host immune reactivity in favor of Th1 responses (IL-12 and IFN-γ) [42]. CpG treatment has been shown to rapidly activate T cells, B cells, macrophage proliferation, and secretion of antibodies as well as an array of Th1-associated cytokines [31]. In addition, CpG is speculated to stimulate non-specific innate inflammatory responses to aid in development of antigen specific immunity. Of the three classes of CpG, class-C attains properties intermediated to those in class-A and -B CpG which include B-cell and NK-cell activation, in addition to induction of IFN-α secretion from DCs [27]. 

Our previous studies have demonstrated that of the different classes of CpG, the class-C provided better protection against an acute respiratory murine model of *B. pseudomallei* infection [15], and therefore, a similar model was chosen for analysis in the present study. Also, the BALB/c mouse model was previously optimized in our laboratory for acute respiratory *B. mallei* infection and bioluminescent imaging [38]. BALB/c mice are a good animal model to study infection since *B. mallei* exhibits an organ-tropism, localizing specifically to the lung, liver and spleen [43]. In addition, BALB/c mice are susceptible to aerosol and due to the symptoms exhibited after infection; this becomes a clinical relevant model for vaccine/therapeutic testing. Thus, in this study, class-C CpG ODN was assessed for the ability to protect against *B. mallei* in the acute respiratory model of BALB/c infection. Protection was evaluated by percentage of survival, bacterial burden, real-time bacterial dissemination and lung histopathology. In an attempt to dissect immune processes of protection, treated and untreated groups were further monitored in real time for neutrophil trafficking and quantified for their neutrophil, inflammatory monocyte and macrophage populations. In addition, this study highlights, for the first time, the use of a new multi-modal imaging technique which employs neutrophil-specific, fluorescent imaging agent, to monitor neutrophil trafficking *in vivo* simultaneously with luminescent *B. mallei* dissemination.

CpG treatment has resulted in the prolonged survival in an array of animal models infected with a variety of intracellular and extracellular pathogens such as *B. pseduomallei,B. mallei, Klebsiella pneumoniae Listeria monocytogenes*, *Yersinia pestis, Francisella tularensis*, and mycobacteria [15,16,17,18,21,22,25,39,43,44,45]. This protection is attributed to the rapid and early induction of innate immunity which provides increased resistance through controlling infection and lowering/preventing the dissemination of pathogens to target organs. Consistent with previous findings [15], class-C CpG treatment resulted in increased survival of BALB/c mice (Figure 1) with lowered and/or undetectable levels of *B. mallei* present in target organs (Figure 2). As expected with the selected infective dose (10^4^ CFUs), the majority of PBS treated animals died on day 3 with 0% surviving on day 7 (Figure 1). Although some animals also died around day 3, protective effects of class-C CpG ODN treatment increased survival to 60% in BALB/c mice until the experiment was terminated on day 21. In parallel with the survival study, mice from each treatment group were sacrificed at different time points to assess the effect of class-C CpG treatment on bacterial load in the lungs, liver and spleen (Figure 2). When looking at bacterial burden in all organs, an inverse trend in the mean values is seen between class-C CpG treated and untreated groups as time progressed. From 24 to 72 h, bacterial burden in untreated BALB/c mice increases exponentially, whereas in class-C CpG-treated BALB/c mice the opposite effect is seen, with bacterial burden decreasing exponentially over time with the exception of the 48 h time point in the spleen, where bacterial burden seemed to increase slightly and then, in the case of two animals, decreased to undetectable levels. Consistently, class-C CpG displays the potential to decrease bacterial number by 6- to 7- logs as seen in all organs by 72 h post-infection. Our results indicated that increased survival in BALB/c mice may be attributed to class-C CpG ODNs ability to control infection in the lungs and/or prevent/reducing *B. mallei’s* dissemination to target organs. 

The use of CpG DNA with a variety of vaccines has improved protective immunity in many animal challenge models. CpG act as an immune adjuvant, accelerating and boosting cellular immune and antigen-specific antibody responses independent of the infecting pathogen [15,16,17,18,19,20,21,22,23,24,25,26,27,28,29,31,32,33,34,35,36,37,38,39,42,44,45]. In the absence of this influx, bacteria are able to multiply and disseminate freely until an appropriate immune response can be elicited, as was the case for the PBS-treated BALB/c mice. By the time an immune response was induced, the infection was too advanced, causing animals to eventually succumb to infection. Previous experiments on CpG treatment in models of *B. pseudomallei* infection attribute the rapid trafficking of neutrophils to protection [15]. As the primary antimicrobial effector cell of innate immunity, neutrophils serve as a first line of defense against pathogens that has infiltrated the body [45,46]. When an inflammatory response is initiated, neutrophils leave the circulatory system and migrate to the site of infection for containment and clearance of the invading pathogen. Classically, neutrophils are viewed as phagocytes that ingest microbes and kill them by different mechanisms [45,46]. Further, neutrophils also provide signals that play a role in innate immune system activation and function, which are important for communication with other innate immune cells (*i.e.*, macrophages, DCs) and the adaptive immune system (T cells and B cells) [46,47]. 

Due to these characteristics and previous supporting evidence gathered with *B. pseudomallei* [15], neutrophils were evaluated for their protective capabilities against *B. mallei* infection. To accomplish this, a new technique for monitoring neutrophil trafficking *in vivo* in real time was optimized using a neutrophil-specific, fluorescent imaging agent. This reagent is a Cyanine7-conjugate, PEG-modified hexapeptide that specifically binds the formylpeptide receptor (FPR) of neutrophils [48]. The main novelty that comes from this experiment is the fact that the dye allows real time monitoring of neutrophils activation and trafficking. Because the dye is non-toxic and the fluorescence signal corresponds to neutrophil number, the same animal can be monitored throughout the duration of the experiment, giving semi-quantitative data, which lowers the number of animals needed for an experiment and also negates the need for animal sacrifice when looking at neutrophil localization. 

However, a more in depth look at the cell populations was taken, were the lungs of treatment groups at 0 (pre-infection) and 72 h post-infection were extracted and processed for flow cytometric analysis (Figure 4). In accordance with earlier studies [15] class-C CpG ODN pre-treatment in BALB/c mice resulted in a substantial increase in inflammatory monocytes at time zero *vs.* PBS-treated BALB/c mice (Figure 4C). Neutrophil numbers increased prior to infection due to CpG treatment (Figure 4D), which we had previously observed; although this increase was not statistically different than PBS treated mice. The lack of differences in the number of events could be explained by sample timing. When optimizing the use of the Neutrophil-Specific, Fluorescent Imaging Agent, the highest fluorescence’s intensity was observed 24 h after CpG treatment; thus, the 24 h pre-treatment time point was chosen for this assay. However, in previous studies of acute respiratory models of *B. pseudomallei* infection, where neutrophils numbers were higher in CpG treated mice *vs.* PBS treated mice at time zero, CpG treatment was administered 48 h before sampling. Thus, given our sampling time points, the window of detecting difference in the neutrophil numbers seen previously may have been missed. To assess class-C CpG treatment effect on lung architecture, histopathological lung sections were examined at 24, 48 and 72 h post-infection (Figure 5). In general, both treatment groups show the same types of inflammatory processes in the lung; although in the PBS treatment animals the pathology seems to be more severe (Figure 5A–C). At 24 h, lungs from class-C CpG ODN- and PBS-treated BALB/c mice (Figure 5A,D) show mild to moderate and moderate to focally severe, respectively, perivascular and peribronchial inflammation with a considerable neutrophilic component (supplemental Figure 1B). Interesting trends are noticed in both treatments. In the PBS-treatment, higher numbers of microabscesses are observed in the lungs at 48 h compared to 24h and 72 h. On the other hand, in class-C CpG ODN treated BALB/c mice; the highest number of microabscesses is observed at 24 h and progressively decreases at 48 and 72 h. This is supported by the early recruitment of immune cells which kill and contain the infection early and thus negates the constant stimulation and influx of inflammatory cells [49]. Further, this would explain the higher number of clear air-sacs, reduction in microabscesses and inflammatory cells and tissue damage in class-C CpG ODN animals.

## 3. Experimental Section

### 3.1. Bacterial Strain

*B. mallei lux* (CSM001), a previously constructed luminescent reporter strain [37], was cultured on Luria-Bertani agar plates supplemented with 4% glycerol (LBG) and 50 µg/ml of Kanamycin (Km) for 48 h at 37 °C followed by an overnight culture at 37 °C with shaking to obtain an exponential growth phase inoculum. Optical density readings (OD_600_) were used to calculate bacterial concentration (CFU/ml). Bacterial colonies sub-cultured in LBG broth were centrifuged, washed and re-suspended in sterile 1X phosphate-buffered saline to obtain the desired infectious dose (1 × 10^4^ cfu). All manipulations of *B. mallei* were conducted in CDC/USDA-approved and registered biosafety level 3 (BSL3) facilities at the University of Texas Medical Branch and experiments with select agents were performed in accordance with BSL3 standard operating practices.

### 3.2. Mice

Female, 6- to 8-week-old, BALB/c mice obtained from Harlan Laboratories (Indianapolis, IN, USA) were housed in microisolator cages under pathogen-free conditions. Animals were provided with rodent feed, water *ad libitum* and maintained on 12 h light cycle. Before experiments, the mice were afforded an adaption period of at least 1 week. Animal studies were performed in accordance with the Institutional Animal Care and Use Committee’s guidelines at UTMB as recommended by the National Institute of Health.

### 3.3. Ethics Statement

This study was carried out in strict accordance with the recommendations in the Guide for the Care and Use of Laboratory Animals of the National Institutes of Health. The protocol was approved by the Animal Care and Use Committee of the University of Texas Medical Branch (Protocol Number: 0503014A).

### 3.4. Survival Studies

Phosphorothioate-stabilized class-C CpG ODNs was purchased from InvivoGen, San Diego, CA, USA. BALB/c mice (*n =* 20) were anesthetized with 3% isoflurane in an oxygen filled induction chamber and then treated intranasal (i.n.) with either 20 µg of CpG class-C 2395 (TCG TCG TTT TCG GCG CGC GCC G) (*n =* 10) or with 1X PBS (*n =* 10) in a 50 µL volume (25 µL/nare). After 24 h post-treatment, BALB/c mice were then challenged i.n. with 10^4^ cfu of *B. mallei* CSM001 in a 50 µL volume (25 µL/nare), a dose empirically determined to show luminescent signal 48 h after CSM001 infection [38]. Animal survival was recorded over a period of 21 days.

### 3.5. Bacterial Burden

BALB/c mice (*n =* 18) were treated with either CpG (*n =* 9) or PBS (*n =* 9) and then challenged with CSM001 as described above. At 24, 48 and 72 h post infection, following whole-body imaging, BALB/c mice (*n =* 3) from each group were sacrificed and harvested for lungs, livers and spleens. Organs were homogenized by fine mincing with surgical scissors followed by pushing through a 70 µm pore nylon tissue strainer. The homogenates were then serial diluted 10-fold for plating on LBG supplemented with 50 µg/ml of Km. Plates were incubated at 37 °C for 48 h prior to cfu determination.

### 3.6. In vivo Imaging

Bioluminescent and fluorescence images were acquired using an IVIS Spectrum (Caliper Corp., Alameda, CA, USA). At 24, 48 and 72 h post-infection, BALB/c mice were anesthetized, placed in an isolation chamber which was then placed in the imaging chamber and connected to the in-chamber anesthesia delivery system and maintained at 1–2% isoflurane. Bioluminescent signal was measured after three minutes exposure with no excitation (filters blocked) and an open emission filter to capture all luminescent signals from labeled bacteria. Three hours prior to imaging, 2nmol of the Neutrophil-Specific, near infrared (NIR) Fluorescent Imaging Agent (Kerafast, Boston, MA, USA) was administered to class-C CpG ODN or PBS-pretreated BALB/c mice by way of tail vein injection. Images were collected after 1 second of exposure utilizing a 745 nm excitation and 800 nm emission filters. To depict the differences in intensity of the signal, bioluminescence and fluorescence are represented in the images with a pseudocolor scale ranging from red (most intense) to violet (least intense) and yellow (most intense) to dark red (least intense), respectively. Scales were manually set to the same values for every comparable image to normalize the intensity of the bioluminescence and fluorescence across time points. Bioluminescent and fluorescent images were than superimposed to show localization of bacteria and neutrophils.

### 3.7. Cell Preparation and Flow Cytometry

BALB/c mice treated with CpG or PBS 24 h prior to *B. mallei* CSM001 challenge as described above were sacrificed at time 0 (right before challenge) and 72 h after infection. Lungs were harvested and prepared as previously described and used for flow cytometric analysis. Briefly, a single cell suspension was prepared by fine mincing the lung tissue with surgical scissors followed by pushing through a 70 µm, followed by a 100 µM, pore nylon tissue strainer (BD Biosciences, San Jose, CA, USA). The resulting homogenate was treated with RBC Lysis Buffer (Sigma) according to manufacturer’s instruction. Trypan blue exclusion was used to determine white blood cell counts and viability. Single cell suspensions were than stained with fluorescent monoclonal antibodies for flow cytometric analysis, using procedures previously described. The following monoclonal antibodies (mAb) against mouse antigens were purchased from BD Biosciences (San Jose, CA, USA), Ly6G/C-PE, CD11c-FITC, CD11b-PerCpCy5.5, and F4/80-APC. Corresponding isotype controls included: IgG2b-PE, IgG2b-FITC, IgG2a-APC, or IgG1-PerCPCy5.5. Cells were then washed and re-suspended in 400 µL of 4% ultrapure formaldehyde (Polysciences Inc.). Samples were fixed for 48 h, with fresh 4% formaldehyde replacement at 24 h, and sterility confirmed by selective plating. A total of 350 µL of sample was analyzed on a FACS Canto (BD Biosciences, UTMB Flow Cytometry and Cell Sorting Core Facility) and compensation for spectral overlap was performed using FACS DIVA software (BD Biosciences). Background fluorescence was determined by isotype- and fluorochrome-matched non-specific control antibodies and analysis was performed using FCS Express v4.0 Flow Research ed. (De Novo Software) as described previously. Data is presented as the number of gated events corresponding to the expected live leukocyte side scatter and forward scatter gate. Assessment is based on the number of F4/80^+^Ly6G/C^+^ (macrophages), F4/80^+^Ly6G/C^+^ (inflammatory monocytes), and F4/80^−^CD11c^−^CD11b^+^Ly6G/C^+^ (neutrophils) cells in the live leukocyte gate. Data are shown as mean ± SEM. One-way ANOVA followed by a Dunnett's multiple comparison tests for group comparisons (GraphPad Software v4.0). Statistically significant values are designated as * *p <* 0.05.

### 3.8. Histopathology of the Lungs

After imaging, BALB/c mice were euthanized and their lungs removed. Lungs were instilled with formalin, paraffin-embedded and processed for histopathology. Hematoxylin and Eosin stained slides were examined by a pathologist (Dr. Sbrana) for differences in inflammation, inflammatory infiltrates, microabscesses and necrosis in the tissues.

## 4. Conclusions

Pre-treatment of CpG 24 h before CSM001 challenge, reduced bacteria burden and tissue damage in target organs which resulted in a 60% increase in protection compared to mock treated animals. This protection can be attributed to an increase in inflammatory monocytes and neutrophils, as illustrated by the successful implementation of a dye that allowed the simultaneous visualization of neutrophils and bacteria. The application of multi-modal imaging could provide a new novel tool for non-invasive monitoring of the dynamics of neutrophils accumulation in the lung over time following induction of infection. This additional capability could prove to be highly valuable as we work toward developing therapeutic interventions for the treatment of *Burkholderia* respiratory infections.
